# Integrated miRNA-/mRNA-Seq of the Habenulo-Interpeduncular Circuit During Acute Nicotine Withdrawal

**DOI:** 10.1038/s41598-020-57907-w

**Published:** 2020-01-21

**Authors:** Alison P. Casserly, Junko Tsuji, Rubing Zhao-Shea, Ciearra B. Smith, Susanna Molas, Andrew R. Tapper, Zhiping Weng, Paul D. Gardner

**Affiliations:** 1 Brudnick Neuropsychiatric Research Institute, Department of Neurobiology, University of Massachusetts Medical School, Massachusetts, USA; 2 M.D./Ph.D. Program, University of Massachusetts Medical School, Massachusetts, USA; 3 Program in Bioinformatics and Integrative Biology, University of Massachusetts Medical School, Massachusetts, USA; 4 Graduate Program in Neuroscience, University of Massachusetts Medical School, Massachusetts, USA

**Keywords:** Molecular neuroscience, Addiction

## Abstract

Tobacco use is the leading preventable cause of mortality in the world. The limited number of smoking cessation aids currently available are minimally effective, highlighting the need for novel therapeutic interventions. We describe a genome-wide approach to identify potential candidates for such interventions. Next-generation sequencing was performed using RNA isolated from the habenulo-interpeduncular circuit of male mice withdrawn from chronic nicotine treatment. This circuit plays a central role in the nicotine withdrawal response. Differentially expressed miRNAs and mRNAs were validated using RT-qPCR. Many of the differentially expressed mRNAs are predicted targets of reciprocally expressed miRNAs. We illustrate the utility of the dataset by demonstrating that knockdown in the interpeduncular nucleus of a differentially expressed mRNA, that encoding profilin 2, is sufficient to induce anxiety-related behavior. Importantly, profilin 2 knockdown in the ventral tegmental area did not affect anxiety behavior. Our data reveal wide-spread changes in gene expression within the habenulo-interpeduncular circuit during nicotine withdrawal. This dataset should prove to be a valuable resource leading to the identification of substrates for the design of innovative smoking cessation aids.

## Introduction

Tobacco use is a prevalent health problem worldwide, with more than 900 million daily smokers^1^ and an estimated 6 million deaths due to tobacco-related illnesses annually^2^. The few pharmacological cessation aids currently available have limited efficacy^3–6^. Without the intervention of new effective treatments, tobacco-related mortality is anticipated to rise to 8 million deaths per year^2^.

The addictive component of tobacco is nicotine, an agonist of nicotinic acetylcholine receptors (nAChRs), ligand-gated ion channels endogenously activated by acetylcholine^7^. nAChRs are enriched in the mesolimbic and habenulo-interpeduncular circuitries involved in nicotine reward and withdrawal, respectively^8–10^. Cessation of nicotine induces a withdrawal syndrome consisting of negative somatic, affective and cognitive symptoms. Affective symptoms include depressed mood, irritability, craving and anxiety^11,12^. While the rewarding properties of nicotine dominate initial drug-taking, it is largely avoidance of affective withdrawal symptoms that promotes relapse and habitual use^13,14^.

The habenulo-interpeduncular circuitry consists primarily of neurons projecting from the medial habenulae (MHb) to the interpeduncular nucleus (IPN) via the fasciculus retroflexus. In mice experiencing spontaneous nicotine withdrawal, there is increased glutamate release from habenular axon terminals, activating GABAergic neurons in the IPN^15^. Activation of the IPN results in the increased somatic signs and anxiety observed during nicotine withdrawal^15,16^. Optogenetic investigation of this circuit has revealed that silencing the MHb decreases activation of IPN neurons and decreases anxiety during nicotine withdrawal^16^.

While much is known about the neurocircuitry responsible for nicotine withdrawal-associated behaviors, the mechanisms underlying the induction of these behavioral alterations are less clear. Chronic drug exposure induces stable neuroadaptations underlying the compulsion for continued use and susceptibility to relapse. These long-lasting changes in the function of the addicted brain are thought to require alterations of gene expression through regulation of transcription factors, epigenetic mechanisms and non-coding RNAs^17,18^.

microRNAs (miRNAs) are non-coding RNA molecules ~22 nucleotides in length that repress the expression of mRNA targets by promoting mRNA decay or translational repression^19^. miRNAs recognize their targets through imperfect base-pairing of the seed region (nucleotides 2–7) with miRNA response elements (MREs) within the 3′-untranslated regions (UTRs) of mRNAs^20,21^. Tobacco smoke and nicotine have been shown to regulate the expression of miRNAs in various cell lines, organisms and tissues^22–26^.

Dysregulation of miRNAs in the brain have been shown to contribute to neurodevelopmental and neuropsychiatric disorders, including drug addiction^27,28^. However, little is known about the role of miRNAs in the mechanisms underlying nicotine dependence and withdrawal. Few studies have examined the regulation of miRNAs by nicotine in specific regions of the mammalian brain^29–31^. Previously, we showed that the expression of miRNAs predicted to target MREs within the 3′-UTRs of nAChR genes was altered after chronic nicotine treatment in whole brain or a specific brain region^30^. Nicotine has been reported to regulate miRNAs and mRNAs in the MHb, with microarrays detecting expression changes in mice self-administering nicotine^31^. However, the targets of these miRNAs and their functions in the context of nicotine dependence have not been elucidated.

Using integrated miRNA- and mRNA-Seq, we asked whether there are changes in miRNA and mRNA expression in the MHb and IPN during acute nicotine withdrawal. We hypothesize that using these sequencing datasets as valuable resources, it will be possible to identify a multitude of miRNAs/genes playing a role(s) in the mechanisms underlying the neuroadaptations involved in nicotine dependence.

## Results

### Differential expression of miRNAs and mRNAs in the habenulo-interpeduncular axis during acute withdrawal from nicotine

**Figure 1:**
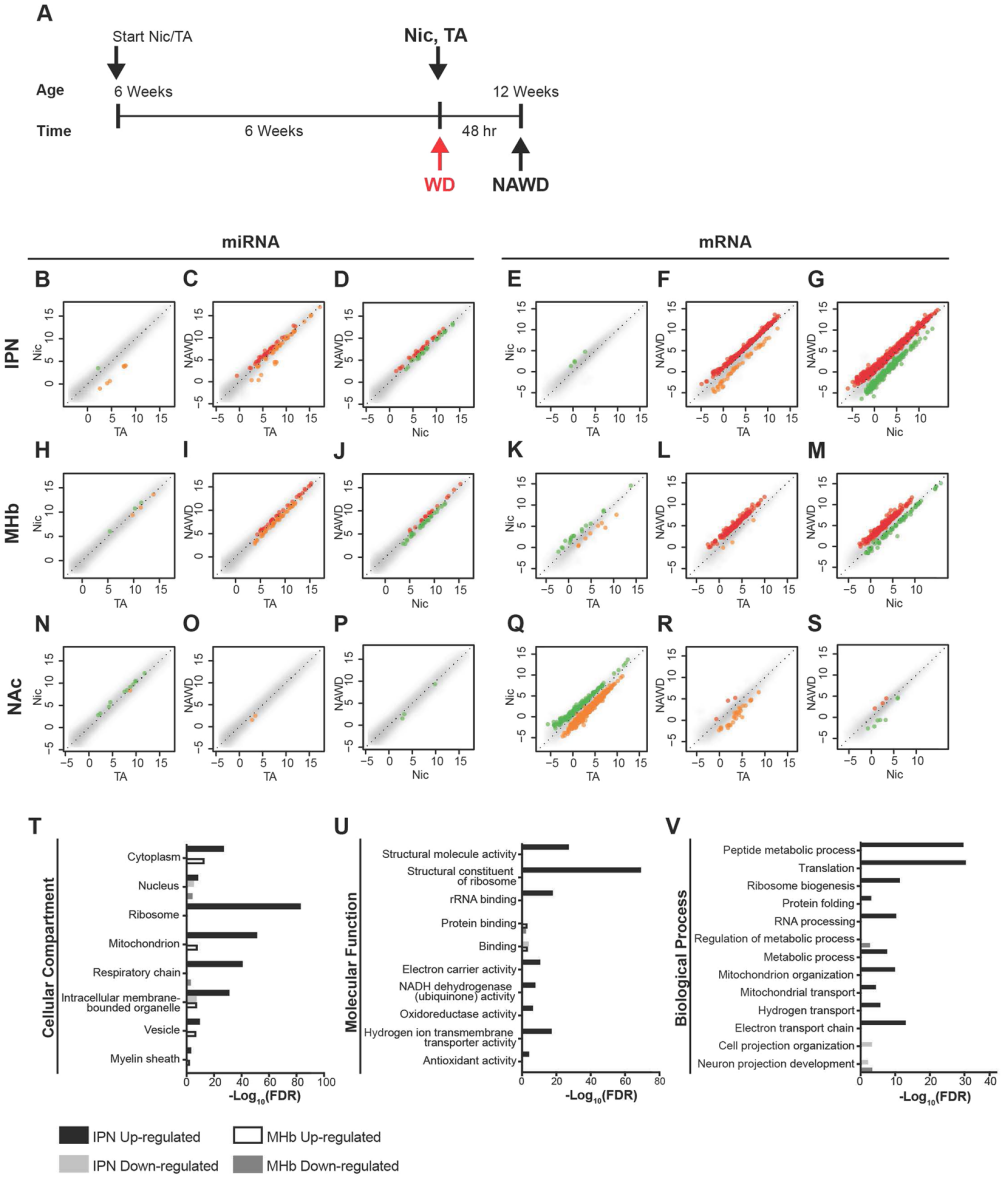
Differential expression of miRNAs and mRNAs within the MHb-IPN withdrawal axis and NAc during acute nicotine withdrawal. (**A**) Schematic of nicotine treatment and spontaneous withdrawal of mice used for miRNA- and mRNA-Seq. Mice were treated chronically with nicotine (Nic) or tartaric acid (TA) for 6 weeks, then either sacrificed or spontaneously withdrawn (WD) for 48 hours before sacrifice (NAWD). (**B–S**) Scatter plots displaying differential expression of miRNAs or mRNAs in the IPN, MHb, and NAc measured by deep sequencing. miRNAs isolated from tissue punches of nicotine-treated (**B,H,N**) and NAWD-treated mice are compared to TA-treated controls (**C,I,O**) or nicotine alone samples (**D,J,P**). mRNAs of nicotine-treated (**E,K,Q**) and NAWD-treated mice are compared to TA-treated controls (**F,L,R**) or nicotine alone samples (**G,M,S**). Each dot represents an individual miRNA or mRNA plotted as log_2_(RPM) or log_2_(TPM), respectively. All miRNAs with an FDR < 0.01 and mRNAs with FDR < 0.01 and FC > 2 are highlighted in color. Orange dots are up-regulated in TA compared to Nic or NAWD. Green dots are up-regulated in Nic compared to TA controls. Red dots are up-regulated in NAWD compared to TA controls. Only miRNAs with RPM > 0 and mRNAs with TPM > 0 in at least half of the replicates in each treatment group are shown. n = 4–5, with each library synthesized using pooled RNA from 4 mice. (**T–V**) Genes differentially expressed during acute nicotine withdrawal are enriched in gene ontology terms related to energetics, translation, and cell projection organization in the IPN and MHb. The vertical axes define the selected GO terms describing the cellular compartment (**T**), molecular function (**U**), or biological process (**V**). The number of GO terms was reduced combining similar terms using a *sim*_*Rel*_ cutoff of 0.4 as described in the Materials and Methods_._ The horizontal axis plots the significance of enrichment of the GO term, plotted as −log_10_(FDR).

To assess the regulation of miRNAs and mRNAs by chronic nicotine treatment (Nic) and acute withdrawal (NAWD), we sequenced and performed differential expression analysis of RNAs isolated from the IPN and MHb (Fig. 1B–M; Figs. S2 and S3). As a reference brain region outside of the habenulo-interpeduncular circuit, we sequenced RNA from the nucleus accumbens (NAc), a prominent mesolimbic reward region (Fig. 1N–S; Figs. S2 and S3).

In the habenulo-interpeduncular circuit, there were few miRNAs differentially expressed after chronic nicotine treatment. In the IPN, only 8 miRNAs were significantly altered after nicotine treatment alone (Fig. 1B). The 7 down-regulated miRNAs remained similarly repressed after a 48-hr withdrawal (see GEO upload). In the MHb, there were only 6 differentially expressed miRNAs in Nic mice compared to TA controls (Fig. 1H), and only 1 remained similarly regulated after nicotine was discontinued (see GEO upload).

However, after acute nicotine withdrawal, the expression of a multitude of miRNAs was altered in the habenulo-interpeduncular circuit. In the IPN of NAWD mice, there were 42 up-regulated and 45 down-regulated miRNAs in NAWD mice compared to TA controls (Fig. 1C). Twenty-nine of these miRNAs were similarly differentially expressed when NAWD mice are compared to either TA or Nic mice (Fig. 1D), suggesting their regulation in the IPN is due entirely to withdrawal and not an effect of nicotine exposure alone.

In the MHb, there were 35 up-regulated and 50 down-regulated miRNAs in NAWD mice compared to TA controls (Fig. 1I). Forty miRNAs were similarly differentially expressed when NAWD mice are compared to either TA or Nic (Fig. 1J), suggesting their expression was altered only after withdrawal, and was not simply an effect of the nicotine treatment.

To determine the persistence of the alterations in miRNA expression following acute nicotine withdrawal, another group of mice underwent a long (4-week) withdrawal after chronic nicotine treatment (NLWD) prior to sequencing. Interestingly, there were no differentially expressed miRNAs in either the IPN or MHb of NLWD mice compared to age-matched TA-treated (TAL) controls (Fig. S2S–V).

In the NAc, expression of 12 miRNAs was altered in Nic mice (Fig. 1N). However, in NAWD mice only 2 miRNAs were differentially expressed (Fig. 1O). Twenty-six miRNAs were differentially expressed in NLWD mice compared to TAL controls (Fig. S2W,X), and none of these were similarly altered in acute withdrawal (see GEO upload).

With respect to the mRNAs, few were regulated by chronic nicotine treatment with a fold change >2 in the IPN and MHb. In the IPN, there were 4 differentially expressed mRNAs in Nic mice compared to controls (Fig. 1E) and all of these returned to control expression levels in NAWD mice (see GEO upload). In the MHb, there were 18 mRNAs up- and 8 mRNAs down-regulated by chronic nicotine treatment compared to TA controls (Fig. 1K) and only 2 remained differentially expressed after acute withdrawal (see GEO upload).

In contrast to chronic nicotine treatment alone, there were 292 up- and 43 down-regulated mRNAs in the IPN of NAWD mice compared to TA controls (Fig. 1F). Of these mRNAs, 303 were similarly altered when NAWD mice are compared to either TA or Nic mice (Fig. 1G), suggesting their regulation was an effect of acute nicotine withdrawal and not an effect of nicotine treatment alone.

In the MHb, there were 151 up- and 3 down-regulated mRNAs in NAWD mice compared to TA controls (Fig. 1L). When NAWD mice were compared to either TA or Nic, the expression of 122 mRNAs was similarly altered (Fig. 1M), suggesting their regulation was an effect of acute withdrawal from nicotine and not an effect of nicotine treatment alone.

In the NAc, in contrast to the habenulo-interpeduncular circuit, there were a total of 852 genes differentially expressed in Nic compared to TA mice (Fig. 1Q). After acute withdrawal, there were only 31 mRNAs differentially expressed in the NAc of NAWD mice compared to TA (Fig. 1R). Of these mRNAs, 25 were also altered after nicotine-treatment alone, suggesting this was largely an effect of the chronic nicotine treatment (Fig. 1S).

To determine the stability of altered mRNA expression induced by nicotine withdrawal, NLWD mice were compared to TAL controls. In the IPN, 39 genes were differentially expressed in NLWD mice compared to TAL (Fig. S3S,T). However, none of these genes were differentially expressed after acute withdrawal (see GEO upload). In the MHb, all mRNAs were expressed at control levels in NLWD mice (Fig. S3U,V).

Gene ontology analysis was performed on differentially expressed mRNAs for all treatment groups and brain regions (Fig. 1T–V, Table S1). For genes up- or down-regulated in the IPN and MHb of NAWD mice compared to TA controls, we determined enrichment of reduced terms describing the cellular compartment (CC, Fig. 1T), molecular function (MF, Fig. 1U), and biological process (BP, Fig. 1V). CC analysis determined there was significant enrichment of genes up-regulated in the IPN related to the mitochondrion. There was also enrichment of MF and BP terms related to mitochondrion, such as electron carrier activity, NADH dehydrogenase activity, hydrogen ion transport, and regulation of metabolic processes. There was also significant enrichment of genes up-regulated in the IPN related to ribosomal terms, including structural constituent of the ribosome, rRNA binding, ribosome biogenesis, and translation. In both the IPN and the MHb there was significant enrichment of down-regulated genes related to neuron projection development.

### Differentially expressed miRNAs are predicted to target reciprocally expressed mRNAs in the IPN and MHb during acute nicotine withdrawal

TargetScan Mouse 7.1 was used to identify transcripts containing conserved MREs potentially targeted by miRNAs in all treatment groups and brain regions (Table S2).

In the IPN, 38 mRNAs that were differentially expressed in NAWD mice compared to controls are predicted targets of at least one of 35 inversely expressed miRNAs (Table 1). For example, expression of *Profilin 2* (*Pfn2*), a regulator of cytoskeletal dynamics enriched in the brain^32^, was down-regulated by ~50% in the IPN of NAWD mice. miR-106b-5p is up-regulated by ~80% and is predicted to target two MREs within the 3′-UTR of *Pfn2*.

**Table 1:** Predicted miRNA targets differentially expressed in the IPN during acute nicotine withdrawal.

mRNA	mRNA FC	mRNA FDR	miRNA	miRNA FC	miRNA FDR	Seed Type	Position in 3′-UTR
**Hbq1a**	3.002	2.57E-07	miR-377-3p	0.592	1.08E-03	7mer-m8	252–258
**Rpp21**	2.852	2.35E-20	miR-136-5p	0.613	9.18E-06	8mer	3537–3544
**Gchfr**	2.696	9.55E-06	miR-129-1-3p	0.570	4.18E-04	7mer-m8	298–304
			miR-129-2-3p	0.590	1.62E-05	7mer-m8	298–304
**Gm20390**	2.669	2.25E-23	miR-429-3p	0.329	3.53E-12	7mer-m8	3377–3383
			miR-429-3p	0.329	3.53E-12	8mer	4329–4336
			miR-429-3p	0.329	3.53E-12	8mer	4714–4721
			miR-200b-3p	0.375	6.58E-08	7mer-m8	3377–3383
			miR-200b-3p	0.375	6.58E-08	8mer	4329–4336
			miR-200b-3p	0.375	6.58E-08	8mer	4714–4721
			miR-344d-3p	0.741	4.47E-03	7mer-A1	3316–3122
**Gm9726**	2.627	2.76E-04	miR-29b-3p	0.726	4.41E-03	8mer	326–333
**Hist1h4h**	2.505	1.18E-15	miR-9-5p	0.753	5.60E-03	7mer-m8	26–32
**Serf2**	2.421	7.81E-20	miR-200a-3p	0.381	1.24E-13	7mer-m8	213–219
			miR-141-3p	0.458	3.07E-04	7mer-m8	213–219
			let-7f-5p	0.735	8.04E-04	8mer	2110–2117
			let-7b-5p	0.741	6.40E-03	8mer	2110–2117
			let-7c-5p	0.766	9.52E-03	8mer	2110–2117
**Mrps16**	2.418	2.40E-15	miR-136-5p	0.613	9.18E-06	7mer-m8	86–92
**Zfp46**	2.409	3.91E-12	miR-129-1-3p	0.570	4.18E-04	7mer-m8	464–470
			miR-129-2-3p	0.590	1.62E-05	7mer-m8	464–470
**Ndufb2**	2.368	2.31E-22	miR-429-3p	0.329	3.53E-12	7mer-m8	3896–3902
			miR-200b-3p	0.375	6.58E-08	7mer-m8	3896–3902
**Esrrb**	2.332	7.67E-08	let-7f-5p	0.735	8.04E-04	8mer	617–624
			let-7b-5p	0.741	6.40E-03	8mer	617–624
			miR-485-5p	0.753	4.04E-03	7mer-m8	55–61
			miR-9-5p	0.753	5.60E-03	8mer	2337–2344
			let-7c-5p	0.766	9.52E-03	8mer	617–624
**Polr2f**	2.323	2.02E-26	miR-488-3p	0.752	8.56E-03	7mer-m8	485–491
**Nme1**	2.305	3.32E-21	miR-200a-3p	0.381	1.24E-13	8mer	123–130
			miR-141-3p	0.458	3.07E-04	8mer	123–130
**Gm9844**	2.296	1.27E-04	miR-129-5p	0.721	1.46E-03	8mer	26–33
**Diras1**	2.234	1.93E-12	miR-7b-5p	0.715	1.25E-03	7mer-m8	2200–2206
**Myl6**	2.234	8.56E-15	miR-340-5p	0.741	6.55E-03	7mer-A1	851–857
**Psma2**	2.174	3.46E-21	miR-132-3p	0.726	6.55E-03	7mer-A1	111–117
**Mrpl14**	2.172	2.53E-14	miR-340-5p	0.741	6.55E-03	7mer-A1	2823–2829
**Ptrhd1**	2.170	1.90E-13	miR-181d-5p	0.676	2.00E-04	7mer-m8	2022–2028
			miR-181b-5p	0.680	5.42E-05	7mer-m8	2022–2028
			miR-488-3p	0.752	8.56E-03	8mer	76–83
**Dynlt1f**	2.169	1.11E-08	miR-29b-3p	0.726	4.41E-03	7mer-A1	215–221
**Sod1**	2.162	7.65E-13	miR-377-3p	0.592	1.08E-03	7mer-m8	78–84
**Cst6**	2.158	6.60E-14	miR-377-3p	0.592	1.08E-03	7mer-m8	2027–2033
			miR-181d-5p	0.676	2.00E-04	7mer-m8	2023–2029
			miR-181b-5p	0.680	5.42E-05	7mer-m8	2023–2029
**Rpl31**	2.153	6.45E-09	miR-653-5p	0.616	4.04E-03	7mer-A1	639–645
			miR-129-5p	0.721	1.46E-03	8mer	671–678
**Tmsb4x**	2.126	2.94E-14	miR-183-5p	0.319	3.96E-21	7mer-m8	327–333
**Ndufb9**	2.104	7.81E-20	miR-200a-3p	0.381	1.24E-13	8mer	2453–2460
			miR-141-3p	0.458	3.07E-04	8mer	2453–2460
			miR-365-3p	0.654	9.94E-03	7mer-m8	2718–2724
			miR-532-5p	0.740	2.06E-03	7mer-m8	3048–3054
			miR-340-5p	0.741	6.55E-03	7mer-A1	1850–1856
			miR-379-5p	0.752	6.55E-03	7mer-m8	3186–3192
**Rps12**	2.097	2.19E-06	miR-96-5p	0.235	1.36E-17	7mer-m8	2285–2291
**Acot13**	2.097	2.94E-07	miR-429-3p	0.329	3.53E-12	7mer-m8	4417–423
			miR-200b-3p	0.375	6.58E-08	7mer-m8	4417–423
			miR-653-5p	0.616	4.04E-03	7mer-A1	5139–5145
			miR-181d-5p	0.676	2.00E-04	7mer-m8	4525–4531
			miR-181b-5p	0.680	5.42E-05	7mer-m8	4525–4532
**Aamdc**	2.094	9.88E-15	miR-485-5p	0.753	4.04E-03	7mer-m8	64–70
**Cisd3**	2.093	9.98E-20	miR-9-5p	0.753	5.60E-03	7mer-A1	358–364
**Tmem151b**	2.035	2.86E-07	miR-129-1-3p	0.570	4.18E-04	7mer-m8	2722–2728
			miR-129-2-3p	0.590	1.62E-05	7mer-m8	2722–2728
			miR-181d-5p	0.676	2.00E-04	7mer-m8	2226–2232
			miR-181b-5p	0.680	5.42E-05	7mer-m8	2226–2233
			miR-132-3p	0.726	6.55E-03	8mer	339–346
			miR-29b-3p	0.726	4.41E-03	7mer-m8	2452–2458
			miR-485-5p	0.753	4.04E-03	8mer	2089–2096
**Erh**	2.031	1.69E-09	miR-181d-5p	0.676	2.00E-04	7mer-m8	118–124
			miR-181b-5p	0.680	5.42E-05	7mer-m8	118–124
**Tomm5**	2.011	4.37E-19	miR-151-3p	0.770	6.36E-03	7mer-m8	383–389
**Gosr2**	0.374	1.22E-11	miR-27a-3p	1.421	8.66E-04	8mer	94–101
**Nkx2-1**	0.459	1.19E-03	miR-503-5p	1.581	1.48E-03	7mer-m8	74–80
**Prkag1**	0.488	8.60E-03	miR-34a-5p	1.941	7.79E-08	7mer-m8	42–48
			miR-34b-5p	1.893	1.43E-06	7mer-m8	42–48
**Lurap1l**	0.489	1.18E-06	miR-497a-5p	2.053	3.09E-13	8mer	60–67
			miR-503-5p	1.581	1.48E-03	7mer-A1	61–67
**Pfn2**	0.496	8.36E-09	miR-106b-5p	1.789	9.32E-11	7mer-m8	452–458
			miR-106b-5p	1.789	9.32E-11	7mer-m8	1197–1203
**Zbtb9**	0.496	3.34E-05	miR-497a-5p	2.053	3.09E-13	7mer-m8	42–48
			miR-497a-5p	2.053	3.09E-13	7mer-m8	45–51
			miR-106b-5p	1.789	9.32E-11	8mer	822–829

TargetScan Mouse 7.1 predicted conserved MREs within the 3′-UTRs of a subset of mRNAs differentially expressed in the IPN of NAWD mice compared to TA controls. miRNAs conserved in vertebrates and mammals that were inversely expressed (FDR < 0.01) and predicted to target these MREs are listed. Only mRNAs with a FC > 2 and an FDR < 0.01 were included in target prediction analysis. Seed type of the predicted MRE and its position within the 3′-UTR are listed.

In the MHb, 50 mRNAs that were differentially expressed in NAWD mice compared to TA controls are predicted targets of at least one of 32 inversely expressed miRNAs (Table 2). The up-regulation of at least four mRNAs (*Arf5*, *Atg7*, *Bcl2l1*, and *Eif5a*) that are predicted to be targeted by down-regulated miRNAs (Table 2), was verified by RT-qPCR (Fig. S4D–G).

**Table 2:** Predicted miRNA targets differentially expressed in the MHb during acute nicotine withdrawal.

mRNA	mRNA FC	mRNA FDR	miRNA	miRNA FC	miRNA FDR	Seed Type	Position in 3′-UTR
**Arf5**	4.543	1.01E-24	miR-29b-3p	0.650	4.95E-06	8mer	392–399
**Pcolce2**	4.447	9.69E-19	miR-340-5p	0.618	2.19E-10	7mer-A1	329–335
			miR-146b-5p	0.721	1.68E-04	7mer-A1	3039–3045
**Tom1l1**	4.011	2.74E-21	miR-340-5p	0.618	2.19E-10	7mer-A1	397–403
			miR-340-5p	0.618	2.19E-10	8mer	2622–2629
			miR-181b-5p	0.763	2.71E-03	8mer	371–378
			miR-142a-5p	0.765	8.25E-03	7mer-A1	2622–2628
			miR-181c-5p	0.770	3.26E-03	8mer	371–378
**Rasd1**	3.629	3.25E-11	miR-377-3p	0.558	4.71E-04	7mer-m8	572–578
			miR-30c-5p	0.710	2.01E-04	8mer	465-–472
			miR-384-5p	0.735	3.14E-04	8mer	465–472
**Pcna**	3.195	6.07E-16	miR-137-3p	0.652	9.24E-08	8mer	420–427
**Spns1**	3.031	1.55E-11	miR-29b-3p	0.650	4.95E-06	8mer	266–273
**Ppp2cb**	3.022	5.91E-20	miR-183-5p	0.551	2.97E-06	8mer	152–159
			miR-142a-5p	0.765	8.25E-03	7mer-A1	142–148
**Steap3**	2.857	2.10E-06	miR-377-3p	0.558	4.71E-04	7mer-A1	704–710
**Dnajc9**	2.787	7.78E-14	miR-132-3p	0.710	1.32E-04	7mer-m8	1040–1046
**Rpa1**	2.704	2.98E-15	miR-30c-5p	0.710	2.01E-04	7mer-m8	1195–1201
			miR-384-5p	0.735	3.14E-04	7mer-m8	1195–1201
			miR-361-5p	0.762	2.72E-03	7mer-m8	591–597
			miR-361-5p	0.762	2.72E-03	7mer-A1	627–633
**Net1**	2.675	4.10E-07	miR-340-5p	0.618	2.19E-10	7mer-A1	31–37
			miR-135a-5p	0.631	7.96E-09	8mer	831–838
			miR-135b-5p	0.645	4.36E-05	8mer	831–838
**Gpx4**	2.617	1.68E-17	miR-374b-5p	0.549	2.62E-05	7mer-A1	833–839
**Ogfod2**	2.613	3.97E-11	miR-340-5p	0.618	2.19E-10	493-500	8mer
**Sin3a**	2.598	2.68E-09	miR-211-5p	0.492	7.97E-12	7mer-m8	870–876
			miR-183-5p	0.551	2.97E-06	7mer-m8	644–650
			miR-493-5p	0.699	3.98E-03	7mer-m8	724–730
			miR-493-5p	0.699	3.98E-03	8mer	840–847
**Eif5a**	2.561	5.52E-19	miR-495-3p	0.472	8.00E-16	7mer-A1	589–595
**Gosr2**	2.511	5.84E-16	miR-493-5p	0.699	3.98E-03	8mer	1649–1656
**Chst15**	2.455	2.63E-07	miR-342-3p	0.704	8.42E-05	8mer	1364–1371
**Tmem159**	2.443	1.65E-04	miR-135a-5p	0.631	7.96E-09	7mer-m8	607–613
			miR-135b-5p	0.645	4.36E-05	7mer-m8	607–613
**Rab40b**	2.414	3.16E-05	miR-211-5p	0.492	7.97E-12	8mer	336–343
**Rabl6**	2.413	2.15E-08	miR-30c-5p	0.710	2.01E-04	8mer	371–378
			miR-384-5p	0.735	3.14E-04	8mer	371–378
**Id3**	2.400	5.82E-11	miR-340-5p	0.618	2.19E-10	7mer-A1	489–495
**Htra2**	2.381	6.59E-05	miR-410-3p	0.667	4.11E-07	7mer-A1	160–166
**Kctd3**	2.355	2.23E-08	miR-29b-3p	0.650	4.95E-06	7mer-A1	432–438
			miR-30c-5p	0.710	2.01E-04	7mer-m8	411–417
			miR-384-5p	0.735	3.14E-04	7mer-m8	411–417
**Atg16l1**	2.346	3.04E-12	let-7k	0.615	1.81E-04	7mer-A1	235–241
			miR-410-3p	0.667	4.11E-07	8mer	1100–1107
			miR-142a-5p	0.765	8.25E-03	7mer-m8	872–878
**Srl**	2.344	8.98E-04	miR-211-5p	0.492	7.97E-12	8mer	2918–2925
			miR-136-5p	0.582	2.87E-08	8mer	818–825
			miR-136-5p	0.582	2.87E-08	8mer	4468–4475
**Marcksl1**	2.271	1.11E-08	miR-23b-3p	0.740	1.75E-03	8mer	576–583
			miR-23a-3p	0.755	3.09E-03	8mer	576–583
**Ccdc32**	2.241	2.68E-07	miR-377-3p	0.558	4.71E-04	7mer-m8	165–171
**Prpf19**	2.227	2.45E-17	miR-377-3p	0.558	4.71E-04	8mer	2354–2361
			miR-421-3p	0.701	6.33E-03	8mer	2841–2848
			miR-203-3p	0.732	7.63E-04	7mer-m8	120–126
**Tbc1d8b**	2.222	2.86E-03	miR-340-5p	0.618	2.19E-10	8mer	2336–2343
			miR-142a-5p	0.765	8.25E-03	7mer-m8	1016–1022
			miR-142a-5p	0.765	8.25E-03	7mer-A1	2336–2341
**Kdelr1**	2.222	7.47E-08	miR-340-5p	0.618	2.19E-10	7mer-A1	313–319
			miR-335-5p	0.637	4.58E-06	7mer-A1	715–721
**Gm2a**	2.184	1.22E-07	miR-6395	0.682	5.46E-03	8mer	591–598
**Zfp622**	2.170	1.71E-11	miR-181b-5p	0.763	2.71E-03	8mer	410–417
			miR-181c-5p	0.770	3.26E-03	8mer	410–417
**Gap43**	2.163	3.62E-03	miR-23b-3p	0.740	1.75E-03	7mer-m8	433–439
			miR-23a-3p	0.755	3.09E-03	7mer-m8	433–439
**Grasp**	2.157	9.32E-04	miR-132-3p	0.710	1.32E-04	7mer-m8	668–674
**Gpr182**	2.134	4.61E-03	miR-30c-5p	0.710	2.01E-04	7mer-A1	2658–2664
			miR-384-5p	0.735	3.14E-04	7mer-A1	2658–2664
**Metap1**	2.125	1.20E-06	miR-211-5p	0.492	7.97E-12	7mer-m8	1354–1360
			miR-382-3p	0.528	3.46E-11	7mer-m8	1373–1379
			miR-181b-5p	0.763	2.71E-03	7mer-m8	1334–1340
			miR-142a-5p	0.765	8.25E-03	7mer-m8	1221–1227
			miR-181c-5p	0.770	3.26E-03	7mer-m8	1334–1340
**Chid1**	2.123	5.05E-07	miR-142a-5p	0.765	8.25E-03	7mer-m8	2673–2679
**Tmem184a**	2.121	3.51E-03	miR-137-3p	0.652	9.24E-08	8mer	2889–2896
			miR-410-3p	0.667	4.11E-07	7mer-A1	3706–3712
			miR-493-5p	0.699	3.98E-03	7mer-m8	2883–2889
			miR-487b-3p	0.702	4.08E-04	8mer	3684–3691
**Htr2c**	2.106	2.43E-10	miR-382-3p	0.528	3.46E-11	8mer	1230–1237
			miR-137-3p	0.652	9.24E-08	8mer	2964–2971
			miR-23b-3p	0.740	1.75E-03	8mer	1871–1878
			miR-23a-3p	0.755	3.09E-03	8mer	1871–1878
**Alpl**	2.103	3.66E-06	miR-211-5p	0.492	7.97E-12	8mer	655–662
**Ddah2**	2.093	1.58E-08	miR-29b-3p	0.650	4.95E-06	7mer-m8	69–75
**Zkscan4**	2.087	1.94E-05	miR-29b-3p	0.650	4.95E-06	7mer-A1	390–396
**Atg7**	2.087	1.98E-05	miR-211-5p	0.492	7.97E-12	8mer	1454–1461
			miR-382-3p	0.528	3.46E-11	7mer-m8	1438–1444
			miR-142a-5p	0.765	8.25E-03	7mer-m8	879–885
**Galnt18**	2.083	1.04E-04	miR-340-5p	0.618	2.19E-10	7mer-A1	245–251
**Thg1l**	2.060	1.05E-03	miR-340-5p	0.618	2.19E-10	7mer-A1	280–286
**Slc25a18**	2.045	5.08E-05	let-7k	0.615	1.81E-04	8mer	106–113
**Api5**	2.023	3.22E-09	miR-203-3p	0.732	7.63E-04	8mer	1699–1706
**Bcl2l1**	2.020	8.98E-07	miR-495-3p	0.472	8.00E-16	7mer-A1	1219–1225
			miR-377-3p	0.558	4.71E-04	7mer-m8	1242–1248
			miR-377-3p	0.558	4.71E-04	8mer	1424–1431
			let-7k	0.615	1.81E-04	8mer	947–954
			miR-342	0.704	8.42E-05	7mer-m8	1243–1249
**Cat**	2.014	3.45E-06	miR-23b-3p	0.740	1.75E-03	8mer	793–800
			miR-23a-3p	0.755	3.09E-03	8mer	793–800
**Ccdc80**	2.011	6.43E-03	miR-29b-3p	0.650	4.95E-06	8mer	213–220
			miR-433-3p	0.673	1.45E-05	8mer	1243–1250

TargetScan Mouse 7.1 predicted conserved MREs within the 3′-UTRs of a subset of mRNAs differentially expressed in the MHb of NAWD mice compared to TA controls. miRNAs conserved in vertebrates and mammals that were inversely expressed (FDR < 0.01) and predicted to target these MREs are listed. Only mRNAs with a FC > 2 and an FDR < 0.01 were included in target prediction analysis. Seed type of the predicted MRE and its position within the 3′-UTR are listed.

In the NAc, neither of the miRNAs differentially expressed in NAWD mice compared to TA-treated controls are conserved, and therefore were not considered in target analysis. However, after nicotine treatment alone, there were 204 differentially expressed mRNAs predicted to be targeted by at least one of 8 inversely expressed miRNAs (Table S2).

### Down-regulation of *Pfn2* in the IPN is sufficient to induce anxiety

**Figure 2:**
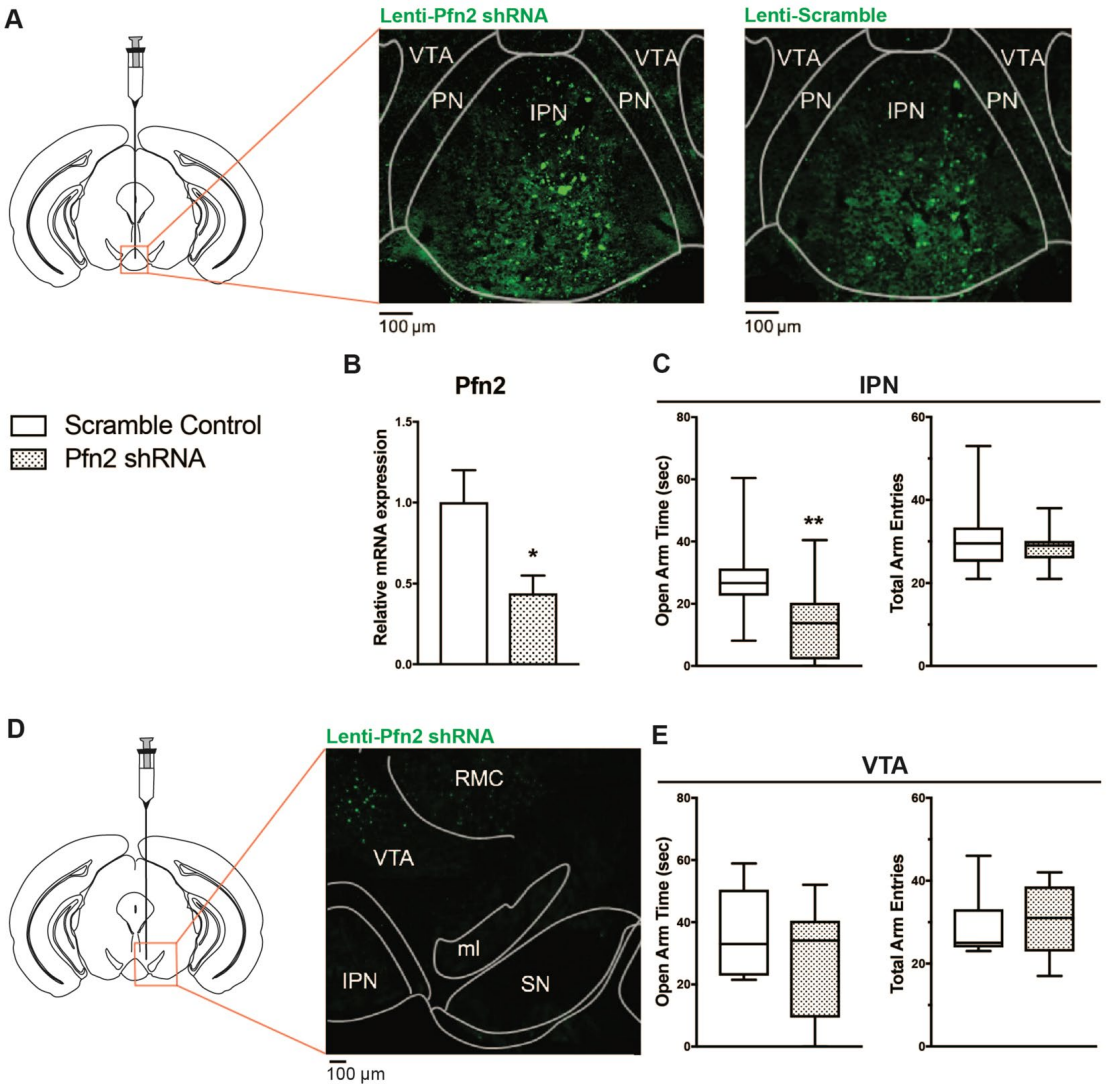
Knockdown of *Pfn2* in the IPN is sufficient to increase anxiety. (**A**) Representative midbrain coronal section schematic and fluorescence microscopy images of WT mouse injected with either lenti-pGIPZ-*Pfn2*-shRNA-tGFP (left image) or lenti-pGIPZ-Scramble-tGFP (right image) in the IPN. Nuclei were labeled with DAPI (blue). (**B**) Knockdown of *Pfn2* expression was measured by RT-qPCR using RNA from laser-captured IPN tissue infected with lenti-pGIPZ-*Pfn2*-shRNA-tGFP compared to lenti-pGIPZ-scramble-tGFP control. mRNA expression was normalized to β-2-microglobulin. Statistical analysis using an unpaired, two-tailed *t*-test compared lenti-*Pfn2* shRNA mice to lenti-scramble controls (*t*_11_ = 2.272, P < 0.05). ^(*)^P < 0.05; n = 6–7. (**C**) Average time spent in the open arms and total number of arm entries in the elevated plus maze of mice injected with either lenti-*Pfn2* shRNA (stippled bars) or lenti-scramble shRNA control (open bars) in the IPN. Statistical analysis used an unpaired two-tailed *t*-test to compare time spent in open arms (*t*_33_ = 3.583, P = 0.0011) and total number of arm entries (*t*_33_ = 0.7355, P = 0.4672) of lenti-*Pfn2* shRNA mice to lenti-scramble controls. ^(**)^P < 0.01 n = 16–19. Box plot edges are 25^th^ and 75^th^ percentile, central line is the median and whiskers are max and min. (**D**) Representative midbrain coronal section schematic and fluorescence microscopy image of WT mouse injected with lenti-pGIPZ-*Pfn2*-shRNA-tGFP in the VTA. Nuclei were labeled with DAPI (blue). (**E**) Average time spent in the open arms and average total number of arm entries in the elevated plus maze of mice injected with either lenti-*Pfn2* shRNA (stippled bars) or lenti-scramble shRNA control (open bars) in the VTA. Unpaired, two-tailed *t-*test was used for statistical analysis of time spent in open arms (*t*_16_ = 1.09, P = 0.2918) and total arm entries (*t*_16_ = 0.4153, P = 0.6835). n = 9. Box plot edges are 25^th^ and 75^th^ percentile, central line is the median and whiskers are max and min.

Withdrawal from nicotine induces anxiety in mice through activation of the IPN^16^. Using our treatment paradigm, NAWD mice display increased anxiety compared to nicotine- and TA-treated controls, as measured by the marble burying test and EPM (Fig. S4A–C). Because *Pfn2*-/- mice display increased synaptic vesicle exocytosis and hyperactivation of neurons^33^, we hypothesized that repression of *Pfn2* in the IPN is sufficient to induce anxiety, mimicking the behaviors observed in NAWD mice. To knockdown *Pfn2* expression, three different lentiviral shRNAs were tested in SN17 cells, revealing that lenti-pGIPZ-Pfn2-shRNA494369-tGFP produced robust knockdown (~81%) compared to virus expressing a scramble control shRNA (Fig. S5A). Injection of the IPN with lenti-pGIPZ-Pfn2-shRNA494369-tGFP (Fig. 2A) resulted in ~54% knockdown of *Pfn2* expression in areas expressing tGFP compared to animals injected with control virus (Fig. 2B). Virus expression was limited to the IPN and adjacent brain areas, with no spread to distant midbrain regions, as detected by fluorescence microscopy (Fig. S5B). Quite strikingly, when *Pfn2* was knocked down in the IPN, mice spent significantly less time in the open arms of the EPM compared to controls (Fig. 2C). Importantly, there was no significant difference in the number of total arm entries, indicating the results are not confounded by non-specific effects on locomotor activity (Fig. 2C). However, when *Pfn2* was knocked down in the neighboring VTA (Fig. 2D), there was no significant effect on the amount of time spent in the open arms of the EPM or total arm entries (Fig. 2E). Because the IPN is also a prominent brain region in the circuitry underlying novelty preference^34^ and novelty seeking behaviors are altered in *Pfn2* knockout mice^33^, we also tested the effect of *Pfn2* knockdown on object novelty preference. Expression of lenti-pGIPZ-*Pfn2*-shRNA494369-tGFP in the IPN did not significantly affect the time spent exploring familiar and novel objects or the novel object preference ratio compared controls (Fig. S6).

## Discussion

Using RNA sequencing and differential expression analysis, we identified wide-spread changes in both miRNA and mRNA expression in the MHb-IPN withdrawal circuit during acute nicotine withdrawal. In contrast, there were a limited number of miRNAs/mRNAs altered by acute nicotine withdrawal in the NAc, a prominent reward center, suggesting that wide-spread changes in transcription during acute withdrawal occur in a brain region-specific manner, concentrated in the habenulo-interpeduncular circuitry.

In the MHb and IPN, miRNAs and mRNAs that were altered during acute nicotine withdrawal largely returned to control expression levels after a prolonged withdrawal. This is most striking in the MHb where there were no differentially expressed miRNAs or mRNAs 4 weeks after withdrawal. In the IPN, none of the mRNAs differentially expressed after a long withdrawal were similarly altered at the 48-hour withdrawal time point. This suggests that for a subset of genes in the IPN, changes in mRNA expression induced by nicotine withdrawal occur over a longer timescale and persist for at least 4 weeks. This subpopulation of genes stably dysregulated by nicotine treatment and withdrawal may contribute to the persistent susceptibility to relapse that plagues nicotine addicts.

Gene ontology analysis identified significant enrichment of up-regulated genes in the IPN related to ribosome structure and translation, further supporting the body of evidence suggesting that stable neuroadaptations associated with drug addiction require *de novo* protein synthesis^17^. Thus, it is possible that up-regulation of ribosomes and other regulators of translation fulfills the increased demand for protein products in the IPN during nicotine withdrawal. In addition, in the MHb and IPN, there is significant enrichment of up-regulated genes related to the mitochondria. The functions of activated neurons, including maintenance of membrane resting potential, intracellular calcium buffering, and synaptic vesicle recycling, are highly energy-demanding^35,36^. To meet the increased demand, neurons increase oxidative metabolism, but cannot up-regulate glycolysis^37^. The enrichment of up-regulated genes related to mitochondria and oxidative energy production reflect the highly active synapses of the IPN during nicotine withdrawal.

An interesting outcome of our work is the realization that some of the miRNAs we demonstrated to be differentially expressed as a result of nicotine withdrawal have been shown by others to regulate expression of mRNAs encoding cholinergic transcripts. Such miRNAs are referred to as CholinomiRs^38^ and include, for example, miR-132 that targets acetylcholinesterase^39,40^, as well as miR-211 that targets the gene encoding the alpha7 subunit of nAChRs^41^. Given that CholinomiRs function in a combinatorial manner such that their impact is a consequence of interactions between competing miRNAs and targets^38^, our dataset should prove useful in further elucidating the mechanisms underlying changes in cholinergic signaling that occur due to nicotine withdrawal.

As an example of the utility of our RNA-Seq data, we focused upon one example of a gene differentially regulated during acute nicotine withdrawal in the IPN. *Pfn2* is a member of the profilin family of small, actin monomer-binding proteins^42,43^. While *Pfn1* is ubiquitously expressed throughout the body, *Pfn2* expression is highest in the brain^32^ and represents approximately 75% of the profilin in rodent brain^32,44^. In addition to its ability to bind actin monomers, *Pfn2* has a poly-l-proline binding domain allowing it to interact with a plethora of ligands whose functions contribute to synaptic morphology and function^42^. *Pfn2* is expressed pre- and post-synaptically^33^. Pre-synaptically, *Pfn2* can influence synaptic vesicle release through interaction with regulators of membrane trafficking and endocytosis^45,46^. Interestingly, *Pfn2 −/−* mice display higher vesicle exocytosis and hyperactivation of the striatum^33^. Post-synaptically, *Pfn2* accumulates in dendritic spines of activated neurons^47,48^, and plays a role in determining their complexity through the regulation of cytoskeletal dynamics^49,50^. *Pfn2* regulates actin polymerization at the synapse by direct interaction with the WAVE-complex^33^, which in turn activates the Arp2/3 complex to promote actin nucleation. Interestingly, *Arpc3* is a confirmed target of miR-29a/b, which is up-regulated in the mouse brain after acute nicotine injection, regulating dendritic spine morphology^29^. Furthermore, *Pfn2* interacts directly with ROCK, a RhoA-GTPase effector, regulating neuritogenesis in response to physiological stimuli^50^. Interestingly, inhibition of ROCK in the mouse brain induces anxiety^51^. Our GO analysis revealed an enrichment of down-regulated genes related to cellular projection organization and neuron projection development in the IPN during nicotine withdrawal, possibly reflecting changes in neuron morphology regulated by the repression of *Pfn2* expression.

In addition to various roles in mechanisms related to cytoskeletal dynamics underlying synaptic morphology, *Pfn2* can also influence the composition of neurotransmitter receptors at post-synaptic surfaces^42^. For example, *Pfn2* can decrease surface localization of excitatory kainate glutamate receptors through regulation of endocytosis and membrane trafficking^52^. *Pfn2* also interacts with membrane scaffold proteins, such as gephyrin^53^, anchoring receptors to the cytoskeleton.

The synaptic roles of *Pfn2* and its link to behaviors such as anxiety led us to hypothesize that down-regulation of *Pfn2* during acute nicotine withdrawal contributes to the neural activation in the IPN during withdrawal, increasing anxiety-associated behaviors. Supporting this hypothesis, we show that knockdown of *Pfn2* expression in the IPN of nicotine-naïve mice was sufficient to increase anxiety as measured by EPM, mimicking behaviors observed in WT mice during acute nicotine withdrawal. Effects on behavior are specific for anxiety, with no alterations in locomotor activity or object novelty preference. The effect of *Pfn2* knockdown is also specific to the IPN, as knockdown within the neighboring VTA does not increase anxiety. Our results suggest a role for the down-regulation of *Pfn2* in neuroadaptations of synapses induced by acute nicotine withdrawal in the IPN. Importantly, with respect to physiological relevance, *Pfn2* is highly expressed in the IPN (expression data of all miRNAs and mRNAs are included in the GEO database associated with this article). *Pfn2* ranks 180^th^ in abundance (mean = 801.258 transcripts per million) amongst all genes detected in the IPN of at least one control replicate putting it in the top 1% of genes detected in at least one replicate (22,181 genes total). It seems likely that a significant reduction in expression of such a highly expressed gene whose product interacts with the cytoskeleton and proteins that regulate synaptic exo- and endocytosis will substantially impact neuronal function. Future studies should focus on determining the effect of withdrawal-induced repression of *Pfn2* on neural activity and synaptic morphology in the IPN.

Our study uncovered wide-spread changes in gene expression of the habenulo-interpeduncular circuit during acute nicotine withdrawal. The multitude of genes and their predicted regulation by miRNAs represent potential novel mechanisms underlying the neuroadaptations of nicotine dependence. Hence, this dataset is a valuable resource, the use of which may lead to the identification of novel substrates for the design of innovative smoking cessation aids and the treatment of withdrawal symptoms, including anxiety. However, it is important to point out that, in addition to miRNA regulation of target genes, it is possible that the targets themselves regulate miRNA levels by regulating miRNA degradation^19^. This is particularly relevant to the nervous system where it has been shown that target RNA-directed miRNA degradation (TDMD) activity is substantially higher in primary neurons versus non-neuronal cells and established cell lines^54^. Thus, our dataset may also prove useful for deciphering TDMD pathways during withdrawal. However, there are limitations to our dataset that must be considered when analyzing miRNA/mRNA relationships following nicotine withdrawal. For example, some of the miRNAs identified using the mouse brain samples are not conserved in humans, raising obvious questions regarding their potential roles in the human condition. Further, while there are a plethora of miRNA/mRNA pairs conserved in all mammals, there are specific miRNA/mRNA pairs that are primate-specific^38^, which would obviously not be detected in our study utilizing mouse brain samples.

It is also of interest to utilize a similar sequencing strategy using other abused drugs (*e.g*., opioids) to determine whether a similar pattern of miRNA/mRNA regulation occurs in the habenulo-interpeduncular circuit, as it is likely that this circuit is involved in the withdrawal response to other drugs in addition to nicotine. Such a result would inform the rational development of therapeutic interventions.

## Materials and Methods

### Animals and drug treatment

All experiments were conducted in accordance with the guidelines for care and use of laboratory animals provided by the National Research Council as well as with an approved animal protocol from the Institutional Animal Care and Use Committee of the University of Massachusetts Medical School (UMMS). Wild-type (WT) male C57BL/6 J (Jackson) were group-housed and were kept on a 12-h light/dark cycle (lights ON 7 A.M.) with food and water provided *ad libitum*. Mice aged 6 weeks were treated with nicotine tartrate (200 μg/ml nicotine base) or equivalent tartaric acid in their drinking water with saccharine (3 g/L) to increase palatability. After 6 weeks, mice in spontaneous withdrawal groups had nicotine bottles replaced with tartaric acid solution. After a 48-hour (Fig. 1A) or 4-week withdrawal period, mice were tested in behavioral assays or sacrificed for tissue collection.

### Tissue collection and RNA isolation

Fresh frozen brains were coronally sliced (~1 mm) and regions were dissected using a circular tissue punch (0.75–1 mm diameter). Bregma ranges for each region were as follows: NAc: 1.8–0.8 mm; VTA: (−3.1) – (−3.9) mm; IPN: (−3.2) – (−3.9) mm. Punches were expelled in lysis buffer (miRVana miRNA isolation kit, Ambion). Punches from 4 or 2 brains were combined in each sample for sequencing and RT-qPCR experiments, respectively. Unless otherwise stated, total RNA was isolated using the miRVana kit according to manufacturer’s instructions. RNA concentration and A260/280 were assessed using a NanoDrop2000 (ThermoFisher).

### Small RNA library synthesis

Total RNA (approximately 2–9 μg, A260/280 >1.8) was separated on a 15% polyacrylamide, 7 M urea, 1X TBE gel. Small RNA (18–30 nucleotides) was excised and eluted from the gel. Libraries were synthesized using a modified protocol for Illumina TruSeq Small RNA Cloning (April 2014, www.zamorlab.umassmed.edu/protocols/). Briefly, small RNAs were ligated to 3′- and 5′-adapters and cDNA was reverse transcribed by AMV Reverse Transcriptase (NEB). The cDNA libraries were amplified by Accuprime *Pfx* DNA Polymerase (Invitrogen) for 18 PCR cycles using a common primer and a primer containing a 6-nucleotide barcoding index. The libraries were gel-purified on a 2.5% Low-Range Ultra Agarose (BioRad) 1X TBE gel and eluted using a QIAquick Gel Extraction kit (Qiagen). Library sizes were assessed by Fragment Analyzer (Advanced Analytical, AATI). Library concentrations were determined using KAPA Library Quantification Kits (KAPA Biosystems).

#### Adapter sequences

3′-Adapter: 5′-rApp/nnntggaattctcgggtgccaagg/ddC/-3′

5′-Adapter: 5′-guucagaguucuacaguccgacgauc-3′

#### Primers

RT primer: 5′-ccttggcacccgagaattcca-3′

PCR forward primer: 5′-aatgatacggcgaccaccgagatctacacgttcagagttctacagtccga-3′

PCR Index Primers (Barcode):

Index Primer 1: 5′-caagcagaagacggcatacgagatcgtgatgtgactggagttccttggcacccgagaattcca-3′

Index Primer 2: 5′-caagcagaagacggcatacgagatacatcggtgactggagttccttggcacccgagaattcca-3′

Index Primer 3: 5′-caagcagaagacggcatacgagatgcctaagtgactggagttccttggcacccgagaattcca-3′

Index Primer 4: 5′-caagcagaagacggcatacgagattggtcagtgactggagttccttggcacccgagaattcca-3′

Index Primer 5: 5′-caagcagaagacggcatacgagatcactgtgtgactggagttccttggcacccgagaattcca-3′

Index Primer 6: 5′-caagcagaagacggcatacgagatattggcgtgactggagttccttggcacccgagaattcca-3′

Index Primer 7: 5′-caagcagaagacggcatacgagatgatctggtgactggagttccttggcacccgagaattcca-3′

Index Primer 8: 5′-caagcagaagacggcatacgagattcaagtgtgactggagttccttggcacccgagaattcca-3′

Index Primer 9: 5′-caagcagaagacggcatacgagatctgatcgtgactggagttccttggcacccgagaattcca-3′

Index Primer 10: 5′-caagcagaagacggcatacgagataagctagtgactggagttccttggcacccgagaattcca-3′

Index Primer 11: 5′-caagcagaagacggcatacgagatgtagccgtgactggagttccttggcacccgagaattcca-3′

Index Primer 12: 5′-caagcagaagacggcatacgagattacaaggtgactggagttccttggcacccgagaattcca-3′

Index Primer 14: 5′-caagcagaagacggcatacgagatggaactgtgactggagttccttggcacccgagaattcca-3′

Index Primer 15: 5′-caagcagaagacggcatacgagattgacatgtgactggagttccttggcacccgagaattcca-3′

Index Primer 16: 5′-caagcagaagacggcatacgagatggacgggtgactggagttccttggcacccgagaattcca-3′

### Small RNA sequencing and analysis

Small RNA libraries were pooled and subjected to single-end (75 cycles) sequencing using a NextSeq. 500 (Illumina) in the RNA Therapeutics Institute at UMMS. Following sequencing, the 3′-adapter sequence was removed using Cutadapt (version 1.11) with ‘–overlap’ option 7 bp. Reads between 15–27 nucleotides long (Fig. S1) were mapped to the UCSC mouse reference genome (mm10) using Bowtie (version 1.1.2) allowing for no mismatches. miRNA reads ±5 nucleotides from the mature, annotated 5′-start position were counted. Reads mapping to multiple locations on a single mature miRNA were recorded as a single count and reads mapped to multiple mature miRNAs were apportioned by the number of the aligned mature miRNAs. Prior to DESeq. 2, read counts were normalized using RUV-Seq^55^, removing 3–4 factors of variance. In each condition, miRNAs with 0 reads per million (RPM) in more than half the samples were omitted from the analysis. Samples with greater than 1 million mappable reads were used in differential expression (DE) analysis by DESeq. 2 (FDR < 0.01)^56^.

### mRNA library synthesis

RNA >30 nucleotides long was eluted from the same denaturing gel as the small RNA. Libraries were synthesized using the TruSeq Stranded mRNA Library Prep kit (Illumina) according to manufacturer’s instructions. Libraries were gel-purified, assessed by Fragment Analyzer and their concentrations were quantified as described for the small RNA libraries.

### mRNA sequencing and analysis

mRNA libraries were subjected to paired-end (76 cycles x 2) sequencing. Reads were mapped to the UCSC mouse reference genome (mm10) using STAR (version 2.5.3a). Following sequencing, mapped reads were quantified with RSEM (version 1.3.0) and RNA-seq libraries with >30 million mappable reads were used in DE analysis by DESeq. 2 (version 1.14.1; FDR < 0.01 and fold change >2). Prior to the DE analysis, read counts were normalized using RUV-Seq (version 1.8.0)^55^ to remove unwanted variables including batch effects. Genome Ontology (GO) term enrichment for biological processes, molecular function, and cellular component was performed on DE mRNAs using GOseq (version 1.28.0)^57^. REVIGO was used to reduce GO terms by combining closely related terms^58^. To provide a well-balanced representation of reduced GO terms, a *simRel* cutoff of 0.4 was used^59^. Raw and processed RNA-Seq data are available at the Gene Expression Omnibus (GEO) of the National Center for Biotechnology Information (NCBI) of the National Institutes of Health, accession # GSE117069. *Reviewers, please see ‘Data Availability’ section for access token*.

### Quantitative reverse transcription-PCR (RT-qPCR)

RT-qPCR for miRNAs and mRNAs was performed as previously described^30^ using TaqMan assays (ThermoFisher) for miR-106b-5p (Assay ID 000442), *Pfn2* (Assay ID Mm01289572_m1), and Pfn1 (Assay ID Mm00726691_s1). Relative miRNA/gene expression was determined using the 2^−ΔΔCt^ method^60^. miRNA and mRNA expression were normalized to snoRNA202 (Assay ID 001232) and β-glucuronidase (Assay ID Mm01197698_m1), respectively, unless otherwise noted.

### Target prediction analysis

TargetScan Mouse 7.1 (http://www.targetscan.org/mmu_71/) was used to identify DE miRNAs that are predicted to target conserved MREs within the 3′-UTR of inversely expressed mRNAs. Analysis was limited to miRNAs conserved in vertebrates and mammals.

### *In Vitro* Knockdown of Pfn2 with Lenti-pGIPZ-Pfn2-shRNA-tGFP

The mouse septal cholinergic cell line SN17^61^ was used to test distinct shRNAs for their abilities to knock down expression of *Pfn2*. SN17 cells were grown in Dulbecco’s Modified Eagle’s Medium (1X DMEM, Corning) supplemented with 10% fetal bovine serum and 100 U/ml penicillin/streptomycin. The cells were infected with three lots of commercially available lentiviral particles expressing one of three distinct shRNAs directed against *Pfn2* or a scramble shRNA control (Lenti-pGIPZ-shRNA, Dharmacon). The shRNA source clones targeted the following sequences: V3LMM_494366 5′-TTTCCTACAATCATATCTA-3′, V3LMM_494367 5′-TATCCACGTAGCTCTGCCA-3′, V3LMM_494369 5′-CTGATCACAGAACACTTCT-3′. SN17 cells (passage 5) were seeded at a density of 2.5 × 10^4^ cells per well in a 24-well plate and incubated overnight. Cells were transduced with lentivirus (multiplicity of infection 0.4) in technical duplicates using 8 μg/ml polybrene in a total of 250 μl in each well. After 5 hours, an additional 1 ml of complete growth medium was added to each well. Approximately 48 hours after transduction, medium was aspirated and replaced with 0.5 ml of complete growth medium containing 2.5 μg/ml puromycin to select for cells successfully transduced with lentivirus. Puromycin selection was performed for a total of 5 days, changing medium every other day. On day 5, cells were harvested and total RNA was isolated using the RNAqueous Total RNA Isolation Kit (ThermoFisher AM1912), eluting in 60 μl. RNA (125 ng) was reverse transcribed using M-MLV Reverse Transcriptase (ThermoFisher Cat No. 28025013) according to manufacturer’s instructions. Relative expression of *Pfn2* was measured by RT-qPCR as described above.

### shRNA-mediated inhibition of *Pfn2*

To inhibit *Pfn2* expression *in vivo*, we employed lentiviral delivery of shRNA targeting *Pfn2* (sense sequence 5′-AGAAGTGTTCTGTGATCAG-3′) to the IPN or VTA. Lenti-pGIPZ-*Pfn2*-shRNA-tGFP (2.37 × 10^8^ TU/ml, V3LMM_494369, Dharmacon) or a control virus expressing a non-silencing shRNA (lenti-pGIPZ-scramble-tGFP, 9.06 × 10^8^ TU/ml, RHS4348, Dharmacon) were injected into IPN or VTA and expressed for 4 weeks prior to all experiments. Stereotaxic injections were performed under aseptic conditions using mice aged 6 weeks as described by Molas *et al*.^34^. Virus was injected in the IPN or VTA (unilateral) at coordinates measured from Bregma (in mm, anterioposterior, mediolateral, dorsoventral at 0°): IPN (−3.47, ±0, −4.77), VTA (−3.45, ±0.5, −4.2) using a 26 s gauge 10-μl syringe (701RN, Hamilton) to deliver 300 nl of virus at a controlled rate of 60 nl/min.

Fresh frozen brains were cryosectioned (12-μm), fixed in ice-cold acetone, and dehydrated in ethanol (70%, 90%, 100%) and xylenes. Areas expressing tGFP were isolated by laser capture microdissection (LCM; Arcturus). RNA was isolated using RNAqueous-Micro Total RNA Isolation Kit (Ambion) for RT-qPCR.

### Behavioral assays

All behavioral testing was performed during the light phase (8:00 A.M. to 5:00 P.M.) in dim white light after habituation to the testing room (≥30 min). On the first test day, mice underwent the marble burying test followed by the elevated plus maze (EPM) as described by Zhao-Shea *et al*.^16^. Approximately 48 hours later, mice underwent object novelty preference testing. Testing was performed in two cohorts of virus-injected mice, with balanced treatment groups by experimenters blind to virus injection. Lenti-virus expression was confirmed by fluorescence microscopy of tGFP. Mice with no virus expression in the brain region of interest were excluded from analysis.

#### Marble burying

For 2 days prior to testing, mice were habituated to individual standard mouse cages, filled 5–6 cm with bedding material, for approximately 1 hour per day. On the test day, 15 sterilized 1.5-cm diameter glass marbles were placed in the cages, equally spaced in a 3 × 5 grid arrangement. Mice were placed in the test cage for 30 min. then returned to their home cage. The number of marbles buried at least 75% with bedding was counted.

#### Object novelty preference test

A T-shaped maze (three arms, each 9 × 30 × 20 cm, connected through a 9 × 9 cm central zone) made of white Plexiglas was used to examine interest in familiar and novel inanimate objects under dim red light conditions. First, the mouse was habituated to the apparatus for 5 min and removed. Two identical plastic objects were placed in the ends of the T-maze, one in each end. The mouse was immediately placed back into the maze for 5 min and allowed to investigate the two identical novel objects (N_1_). The mouse was removed and one object was replaced by a novel object in the same location as the previous object (counter-balanced). The mouse was immediately placed in the T-maze and allowed to investigate the familiar (F_1_) and novel (N_2_) objects. All sessions were video recorded from above (HDR-CX4440 camera, Sony) and mouse behavior was tracked automatically using EthoVision XT 11.5 (Noldus Apparatus). A blind experimenter manually scored the times the mouse explored the familiar and novel objects. Exploration was defined as sniffing with the nose directed at the object from a distance of less than 2 cm. Simply sitting or resting next to the object was not counted as exploration. The novel object preference ratio was calculated as: (total novel object investigation – total investigation)/(total investigation) over the third 5-min session. The apparatus and objects were cleaned with Micro-90 solution (International Products Corporation) to eliminate olfactory traces after each session.

## Supplementary information

Supplementary Information.

## Data Availability

Raw and processed RNA-Seq data are available at the Gene Expression Omnibus (GEO) of the National Center for Biotechnology Information (NCBI) of the National Institutes of Health, accession # GSE117069.
