# Supplementary material for: Integrated miRNA-/mRNA-Seq of the Habenulo-Interpeduncular Circuit During Acute Nicotine Withdrawal
Source: Sci Rep. 2020 Jan 21;10:813. doi: 10.1038/s41598-020-57907-w (PMC6972841; doi:10.1038/s41598-020-57907-w)
Supplement: Supplementary file 1 — Supplementary Information. [file 41598_2020_57907_MOESM1_ESM.docx]

**Supplementary Information**

Integrated miRNA-/mRNA-Seq of the Habenulo-Interpeduncular Circuit During Acute Nicotine Withdrawal

Alison P. Casserly^1,2^, Junko Tsuji^3^, Rubing Zhao-Shea^1^, Ciearra B. Smith^1,4^, Susanna Molas^1^, Andrew R. Tapper^1^, Zhiping Weng^3^ and Paul D. Gardner^1,*^

^1^Brudnick Neuropsychiatric Research Institute, Department of Neurobiology, University of Massachusetts Medical School

^2^M.D./Ph.D. Program, University of Massachusetts Medical School

^3^Program in Bioinformatics and Integrative Biology, University of Massachusetts Medical School

^4^Graduate Program in Neuroscience, University of Massachusetts Medical School

**Supplementary Figures**

**
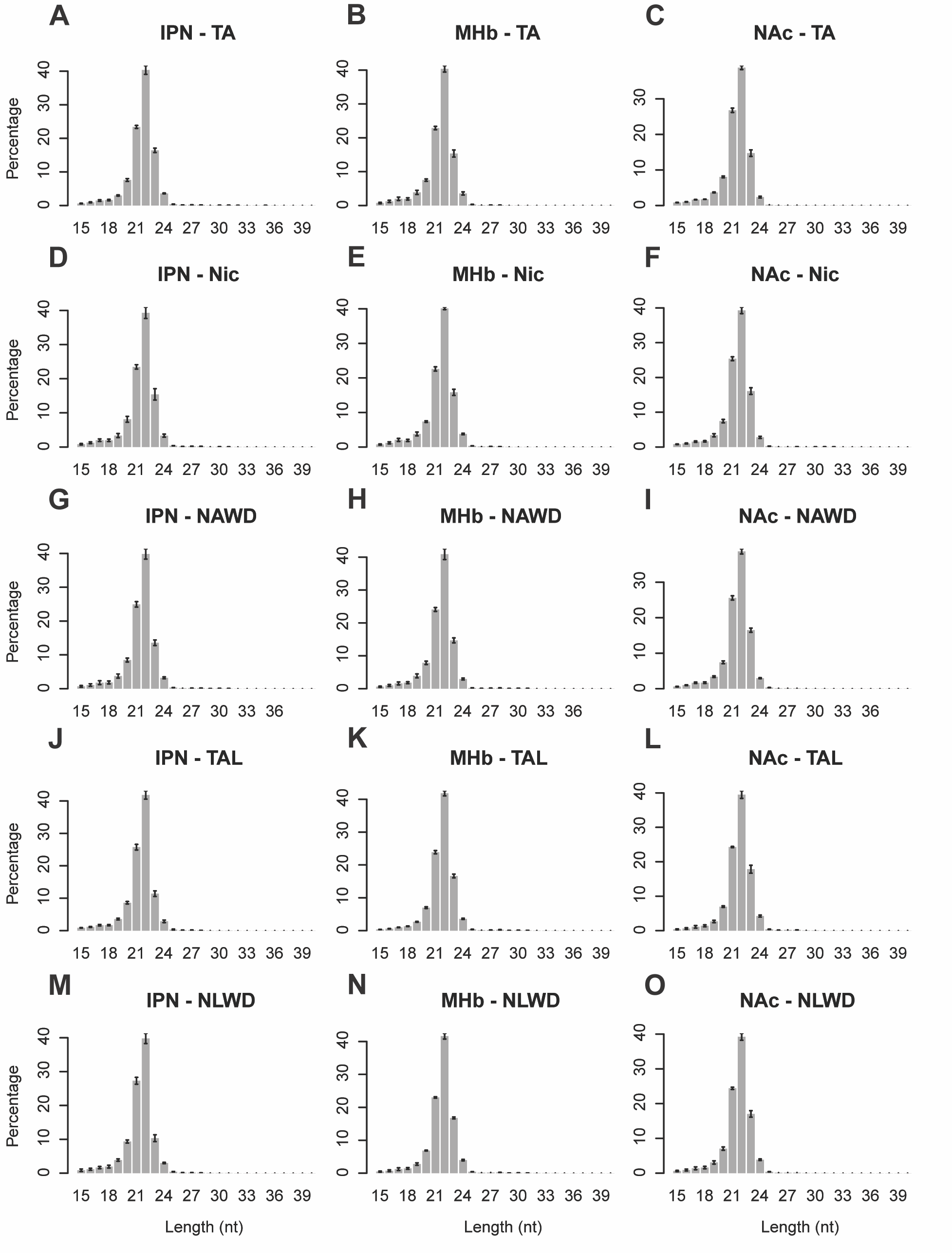
**

**Figure S1. Length distribution of miRNA reads.** To assess the quality of the miRNA-sequencing libraries, the length of miRNA reads (x-axis) was plotted as a percentage of total reads (y-axis) for the IPN, MHb, and NAc of mice treated with (A-C) 6-week tartaric acid (TA), (D-F) nicotine (Nic), (G-I) acute (48-hour) nicotine withdrawal (NAWD), (J-L) long (10-week) tartaric acid (TAL), and (M-O) long (4-week) nicotine withdrawal (NLWD). In all plots, the majority of reads are 22 nt in length. n = 5.


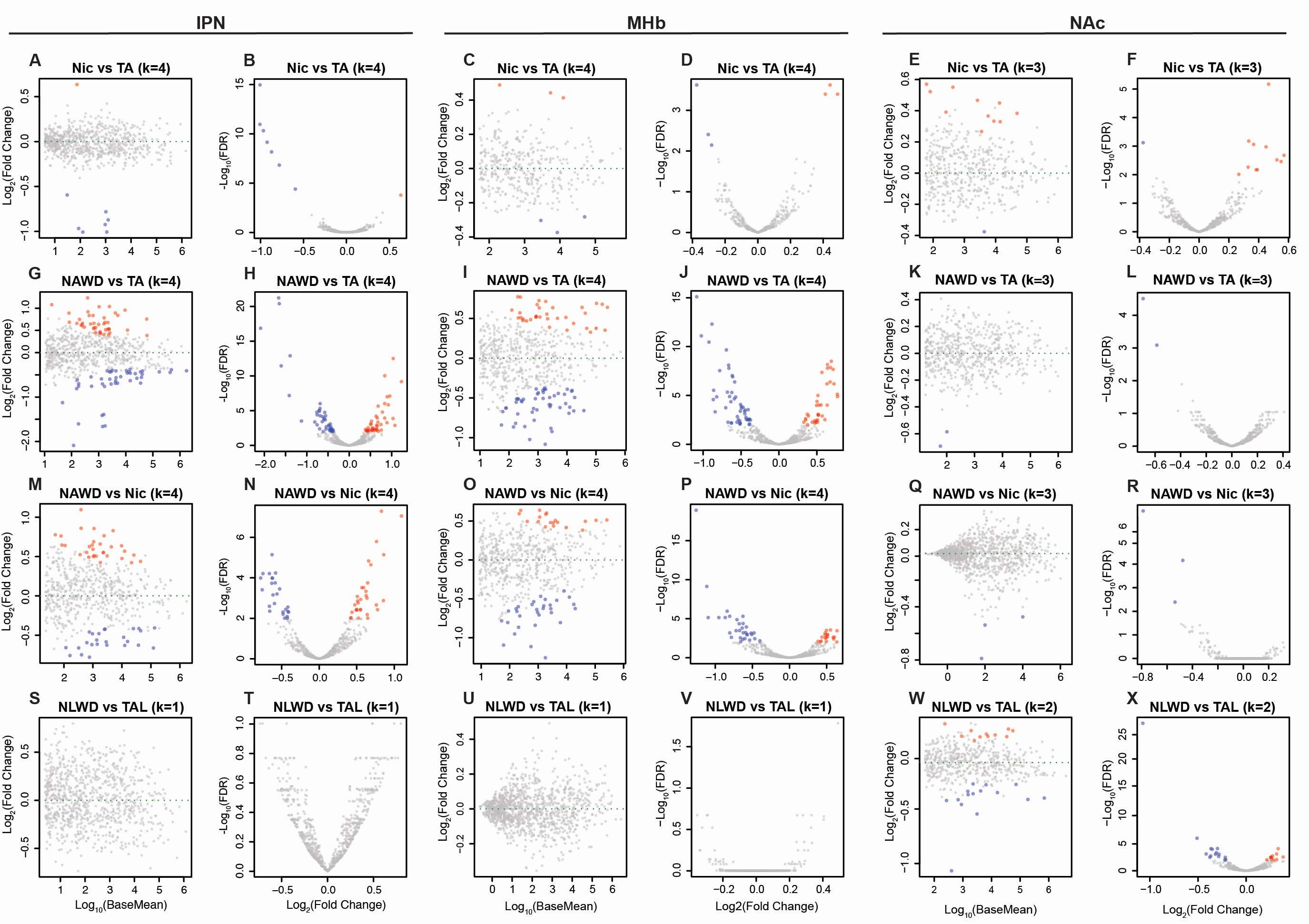


**Figure S2. Differential expression of miRNAs in the IPN, MHb, and NAc of Nic-, NAWD- and NLWD-treated mice.** Differential expression of miRNAs in the three brain regions between different treatment groups was analyzed using DESeq2 as described in the Materials and Methods. The brain region and treatment groups being compared are indicated for each plot. (A-F) Nic-treated mice were compared to TA controls. NAWD mice were compared to TA- (G-L) or Nic-treated (M-R) mice. (S-X) NLWD mice were compared to age-matched TAL control mice (TAL). Each dot in the scatter plots represents an individual miRNA. For each brain region, the left column displays the Log_2_(Fold Change) of the miRNA plotted as a function of the Log_10_(BaseMean). The baseMean is the average number of miRNA counts across all samples in both treatment groups being compared. In the right column, volcano plots display the –Log_10_(FDR) on the y-axis plotted as a function of Log_2_(Fold Change) on the x-axis. In each plot, all up-regulated (red) and down-regulated (blue) miRNAs with FDR < 0.01 are highlighted. The number of factors (k) removed during normalization with RUVSeq is denoted in each plot. n = 5.

**
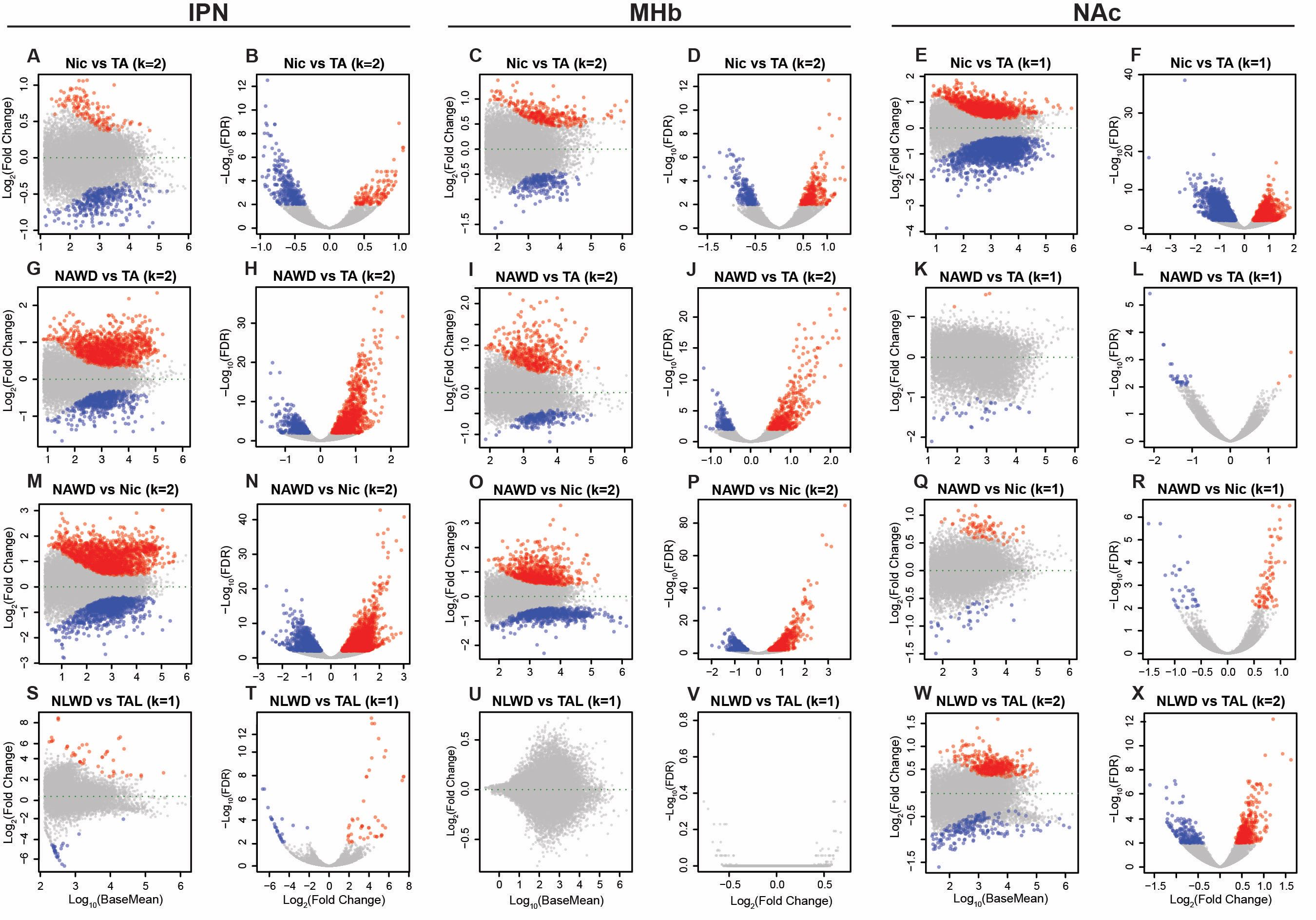
**

**Figure S3. Differential expression of mRNAs in the IPN, MHb, and NAc of Nic-, NAWD-, and NLWD-treated mice.** Differential expression of mRNAs in the three brain regions between different treatment groups was analyzed using DESeq2 as described in the Materials and Methods. The brain region and treatment groups being compared are indicated for each plot. (A-F) Nic-treated mice were compared to TA controls; n = 4. NAWD mice were compared to TA- (G-L) or Nic-treated (M-R) mice; n = 4. (S-X) NLWD mice were compared to age-matched TAL controls (TAL), n = 5. Each dot in the scatter plots represents an individual mRNA. For each brain region, the left column displays the Log_2_(Fold Change) of the mRNA plotted as a function of the Log_10_(BaseMean). The baseMean is the average number of mRNA counts across all samples in both treatment groups. In the right column, the volcano plots display the –Log_10_(FDR) on the y-axis plotted as a function of Log_2_(Fold Change) on the x-axis. In each plot, all up-regulated (red) and down-regulated (blue) miRNAs with FDR < 0.01 are highlighted, regardless of fold change. The number of factors (k) removed during normalization with RUVSeq is denoted in each plot.

**
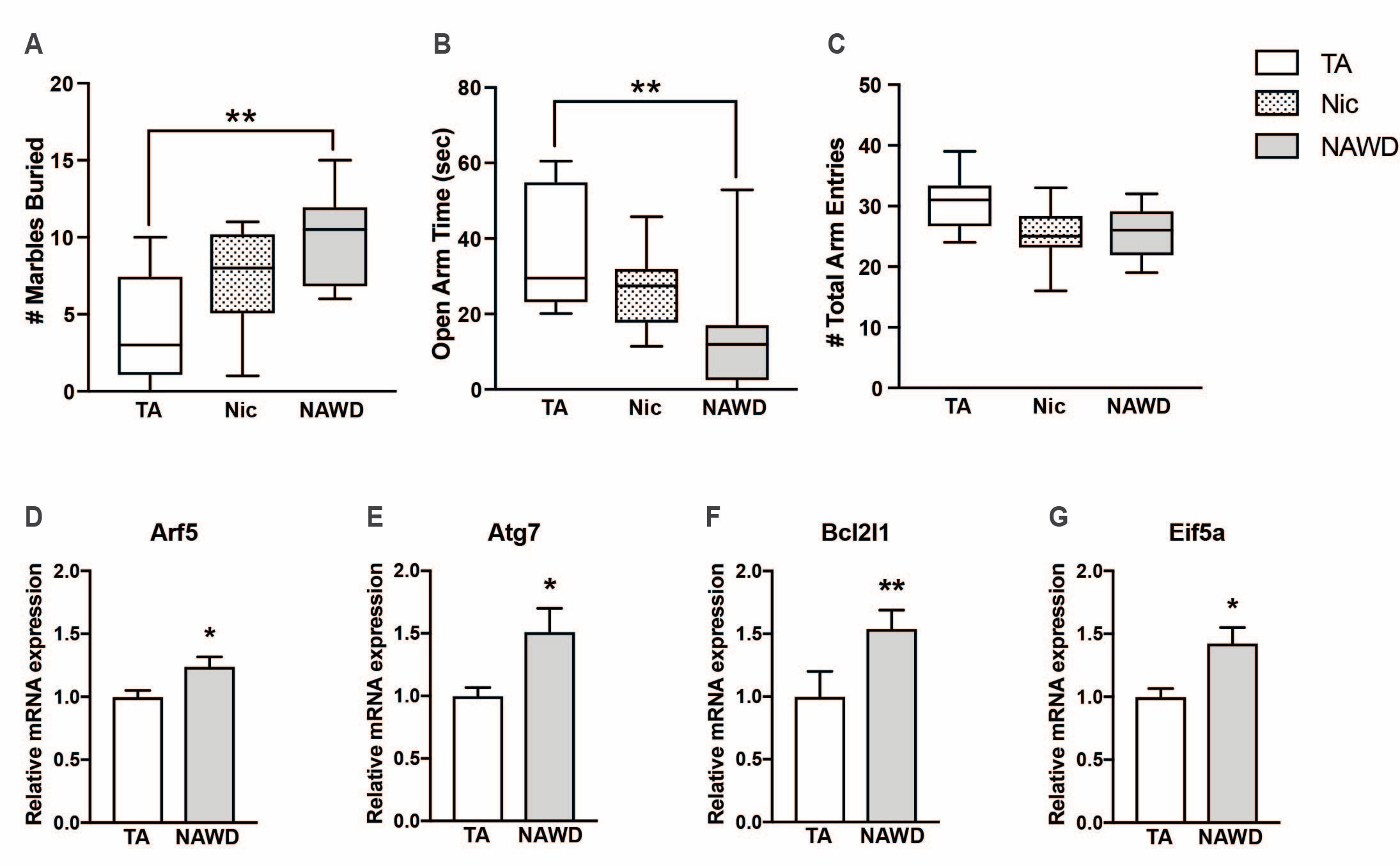
**

**Figure S4. NAWD-treated mice display increased anxiety behaviors and alterations in gene expression.** Anxiety was assayed by the marble burying test (A) and EPM (B, C) to compare mice treated with nicotine (Nic, stippled bars) or acute nicotine withdrawal (NAWD, gray bars) to TA controls (open bars). (A) The number of marbles buried during the marble burying test as described in the Materials and Methods. Statistical analysis by one-way ANOVA (F_2,26_ = 6.974, P = 0.0038) with the Dunnet’s post-test found there were significantly more marbles buried by NAWD mice compared to TA controls. (**) P < 0.01, n = 9-10. Box plot edges are 25^th^ and 75^th^ percentile, central line is the median and whiskers are max and min. (B) Time spent in open arms of the elevated plus maze in 5 min as described in the Materials and Methods. Statistical analysis by one-way ANOVA (F_2,25_ = 5.77, P = 0.0087) with Dunnet’s post-test found NAWD spent significantly less time in the open arms compared to TA controls. (**) P < 0.01, n = 9-10. (C) The number of total arm entries in the elevated plus maze in 5 min. Statistical analysis by one-way ANOVA with Dunnet’s post-test found there were no significant effects on locomotor activity. (D-G) Relative quantitation of genes up-regulated in the MHb during NAWD. RNA was isolated from MHb tissue punches of TA (open bars) and NAWD-treated (gray bars) mice. Relative gene expression levels were measured by RT-qPCR. Data are presented as mean ± SEM, normalized to the TA control. Statistical analysis was performed using unpaired, two-tailed *t*-test to compare expression of *Arf5* (D, *t*_8_ = 2.713, P = 0.0265), *Atg7* (E, *t*_12_ = 2.598, P = 0.0233), *Bcl2l1* (F, *t*_8_ = 4.873, P = 0.0012), and *Eif5a* (G, *t*_8_ = 3.041, P = 0.0160) in NAWD mice compared to TA controls. (*) P < 0.05; (**) P < 0.01; n = 5-7, with each sample containing tissue pooled from 2 mice.

**
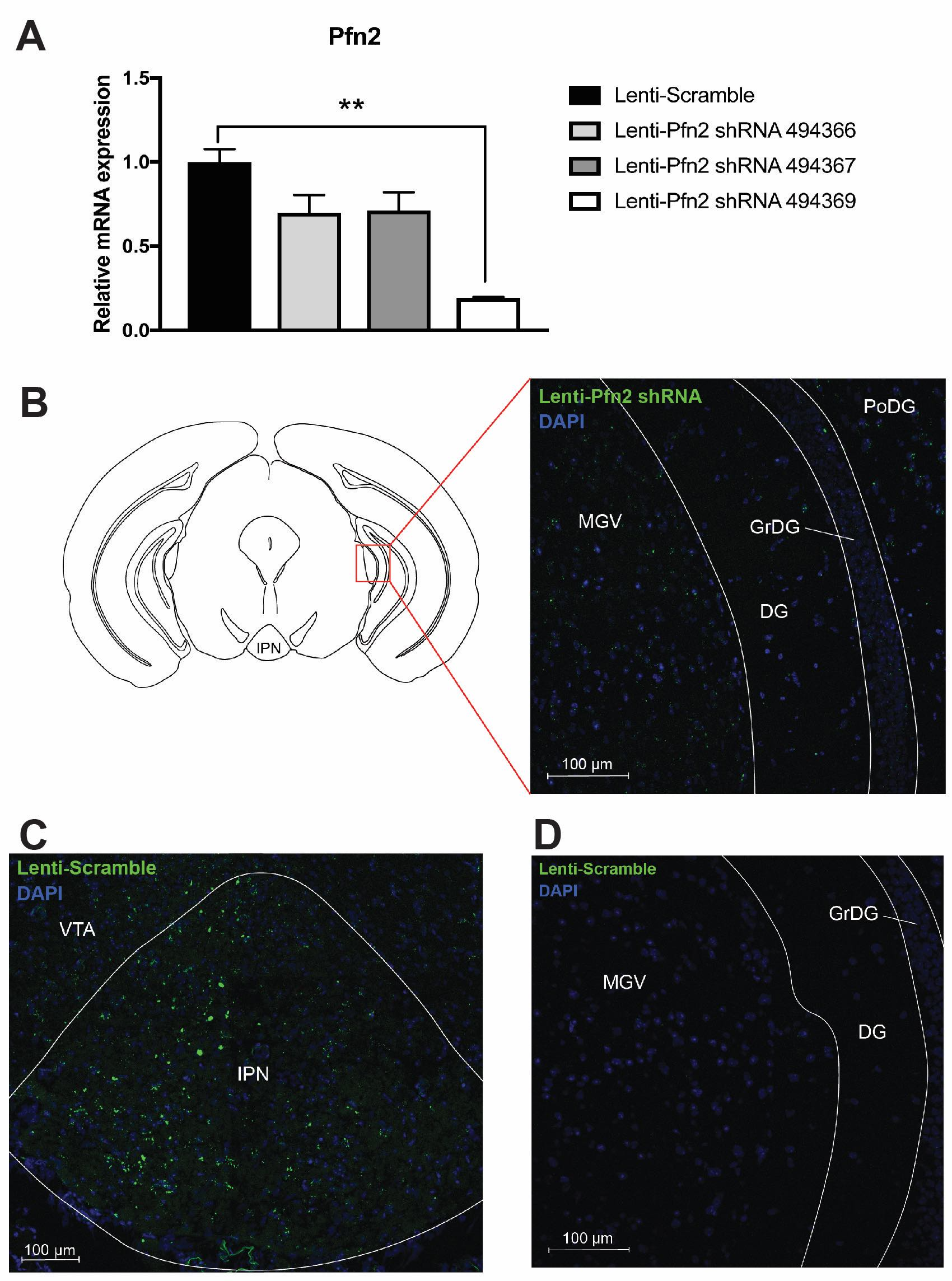
**

**Figure S5. Knockdown of Pfn2 by Lenti-pGIPZ-*Pfn2*-shRNA494369-tGFP.** (A) Transduction of lenti-pGIPZ-*Pfn2*-shRNA494369-tGFP results in approximately 80% knockdown of *Pfn2* in SN17 cells. SN17 cells were transduced with the indicated lenti-pGIPZ-*Pfn2*-shRNA-tGFP or lenti-pGIPZ-Scramble-tGFP negative control as described in Materials and Methods. Total RNA was isolated and relative expression of *Pfn2* was determined by RT-qPCR. Data are presented as mean ± SEM, relative to lenti-scramble control. Statistical analysis was performed using a one-way ANOVA (F_3,4_ = 31.49, P = 0.0031) with Dunnet’s post-test, (**) P < 0.01; (n = 2) (B) Lenti-pGIPZ-*Pfn2*-shRNA-tGFP or Lenti-pGIPZ-Scramble-tGFP injected in the IPN do not spread to distant brain regions within the midbrain. Representative midbrain coronal section from a WT mouse injected with lenti-pGIPz-tGFP (indicated in green) in the IPN. Nuclei were labeled with DAPI (blue). There is no expression of virus above background levels in the MGV of the same slices represented in Fig. 3 of the main text. DG: dentate gyrus; GrDG: granular layer of the dentate gyrus; MGV: medial geniculate complex, ventral part; PoDG: polymorph layer of the dentate gyrus.

**
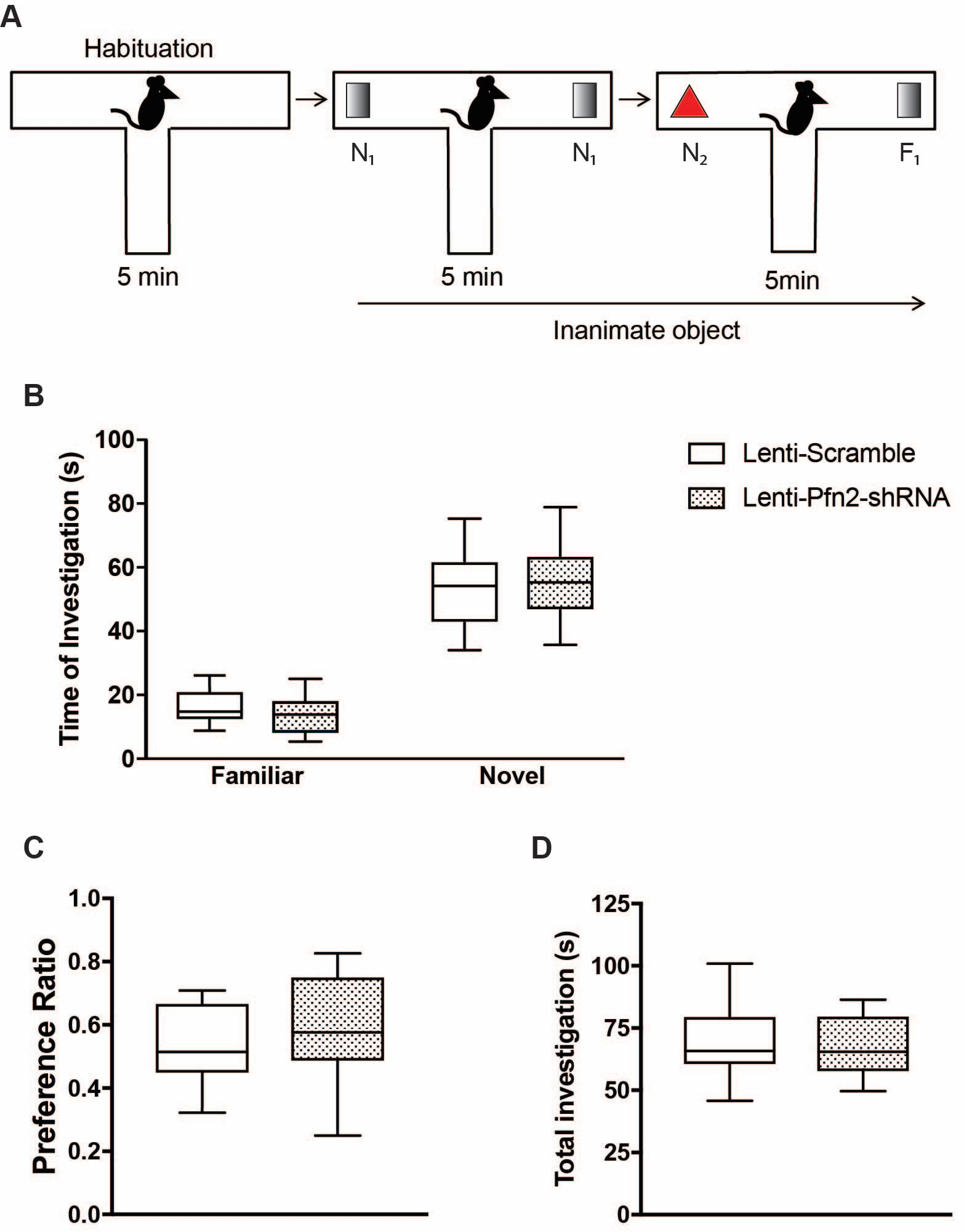
**

**Figure S6. Knockdown of *Pfn2* in the IPN is not sufficient to alter object novelty preference.** (A) Schematic of experimental protocol described in Materials and Methods. (B-D) Mice expressing lenti-pGIPZ-*Pfn2*-shRNA-tGFP (stippled bars) in the IPN were compared to those expressing control virus (open bars). (B) The time of investigation of the familiar (F_1_) and novel (N_2_) objects were manually scored by a blind experimenter for 5 minutes. Statistical testing by two-way ANOVA found there was significant effect of the row factor (object novelty) on the time of investigation (F_1,66_ = 321.2, P < 0.0001). However, there is no significant effect of the column factor (virus injection) on the time of investigation (F_1,66_ = 0.1425, P = 0.7071) (C) There was no difference in the novel object preference (*t*_33_ = 1.039, P = 0.3064, unpaired two-tailed *t*-test). (D) There was no difference in total time investigating the familiar and novel objects combined (*t*_33_ = 0.3692, P = 0.7143, unpaired two-tailed *t*-test). Box plot edges are 25^th^ and 75^th^ percentile, central line is the median and whiskers are max and min.
